# Correlation between SARS-CoV-2-specific antibody titers and the hormones DHEA, cortisol, testosterone, and progesterone

**DOI:** 10.3389/fimmu.2025.1560623

**Published:** 2025-07-31

**Authors:** Tanja Karl, Anja Schuster, Janne Cadamuro, Gertie Janneke Oostingh

**Affiliations:** ^1^ Department of Health Sciences, Salzburg University of Applied Sciences, Salzburg, Austria; ^2^ Research Program of Medical Sciences, Paracelsus Medical University, Salzburg, Austria; ^3^ Department of Laboratory Medicine, Paracelsus Medical University, Salzburg, Austria

**Keywords:** SARS-CoV-2-specific antibodies, COVID-19, hormones, DHEA, cortisol, testosterone, progesterone, personalized medicine

## Abstract

Hormones, such as DHEA, cortisol, testosterone, and progesterone play an important part in the regulation of the human immune system. However, the exact role of endocrine factors in the production of antibodies, in this case SARS-CoV-2-specific antibodies, remains poorly understood. We investigated the association between hormone levels and SARS-CoV-2 spike-protein-specific IgG antibody titers in a large, diverse cohort of 861 vaccinated as well as vaccinated plus COVID-19 recovered individuals. We observed negative correlations between cortisol, progesterone, testosterone (in males), and SARS-CoV-2-specific antibody levels. In contrast, a positive correlation was found between DHEA and antibody titers in vaccinated males. These hormone-antibody relationships exhibited important sex-specific differences. Our findings demonstrate that hormonal factors are associated with modulating the antibody response to SARS-CoV-2, with implications for personalized approaches to vaccination and treatment. Furthermore, the wide variability in hormone levels within the healthy population also suggests the potential value of incorporating endocrine assessments into COVID-19 risk profiling. Further research is needed to fully elucidate the mechanistic underpinnings of these hormone-antibody relationships and explore their broader clinical applications in the context of the ongoing COVID-19 endemic.

## 1 Introduction

The human immune system is complex and finely tuned by various factors, including hormonal influences. Critical hormones modulating immune function are among others dehydroepiandrosterone (DHEA), cortisol, testosterone, and progesterone. These hormones, produced by the adrenal glands and gonads, regulate immune responses by either activating the immune system or suppressing inflammation (3–5). Immune cells have hormone receptors, and circulating hormones can modulate the immune system via these receptors (6).

An improved understanding of how hormones affect immune responses to vaccines or viral diseases is crucial for improving our response to pandemics like the corona virus disease 2019 (COVID-19) pandemic. Epidemiological studies have demonstrated sex-based discrepancies in COVID-19 clinical outcomes, with women experiencing lower infection and hospitalization rates, as well as a better prognosis and mortality (7, 8). Several studies have examined the relationship between specific hormone levels, such as testosterone, estradiol, progesterone, and DHEA, and COVID-19 disease severity (8, 9). However, the link between hormones and the production of severe acute respiratory syndrome coronavirus type 2 (SARS-CoV-2) specific antibodies remains poorly understood. As the antibody response is important for immunity and vaccine efficacy, investigating the role of hormones in modulating this response has significant implications.

DHEA is a precursor to reproductive hormones, and its quantity, similar to the quantity of testosterone, varies with age and sex, complicating its role in the immune response (3, 10). DHEA boosts immunity by increasing interleukin-2 (IL-2) synthesis, increasing T-cell numbers, decreasing the release of inflammatory cytokines, and improving host resistance to viral and bacterial infections (11). Conversely, cortisol has immunosuppressive effects. It inhibits T-cell function and regulates the cellular composition of blood (12). Cortisol levels peak 30–40 minutes after waking and fall during the day (13).

Testosterone, the primary male sex hormone, potentially contributes to sex differences in immune function. It generally suppresses the immune response to bacterial and viral infections by lowering antibody responses to vaccination and infection, decreasing inflammatory immune cells, boosting regulatory immune-like myeloid-derived suppressor cells, and inhibiting T- and B-cell formation and function (1, 2). Progesterone is crucial in pregnancy and immune responses in both sexes. It can reduce inflammatory responses, immune cell activation, and cytokine production (14).

The overarching aim of our project was to identify factors that influence the immune response to SARS-CoV-2 vaccination and/or natural infection, with the goal of enabling more personalized vaccination strategies in future pandemic scenarios. In a previous study, we analyzed several influencing factors and found that vaccine type, vaccine combinations, chronic illnesses, and certain medications significantly affected the immune response to SARS-CoV-2 (15). The present study builds upon these findings with the objective of investigating the correlation between specific hormone levels (DHEA, cortisol, testosterone, and progesterone) and SARS-CoV-2 spike-protein-specific IgG antibodies in a large, diverse study population of 861 subjects. The null hypothesis (H_0_) states that there is no statistically significant association between antibody concentrations and hormone levels. Conversely, the alternative hypothesis (H_1_) suggests that antibody levels are correlated with individual hormone levels within the respective subpopulations. Importantly, this cohort included vaccinated individuals and those with hybrid immunity from infection and vaccination, thereby providing unique insights.

## 2 Materials and methods

### 2.1 Study design and participants

The research cohort comprised 861 individuals aged 19 and older from the Salzburg County, Austria. These individuals had received SARS-CoV-2 vaccinations (Comirnaty and/or Vaxzevria) and/or had encountered COVID-19. The cohort was divided into vaccinated only and hybrid immunized (vaccinated and convalescent) groups.

Participants gave informed consent, which included a data protection declaration. They completed a questionnaire to assess factors potentially impacting antibody levels as previously reported (15). Serological confirmation of the infection status was obtained.

### 2.2 Blood sampling and antibody detection

Blood samples were collected individually between January and June 2022, regardless of vaccination or SARS-CoV-2 infection dates. All blood collections were performed between 9:00 a.m. and 4:00 p.m. A 3 mL serum tube (CAT serum clot activator, ref. no. 454095, Greiner Bio-One GmbH, Austria) was left upright at room temperature for 30 minutes, and thereafter centrifuged at 1300 x g for 10 minutes at 22°C. The resulting sera were transferred into fresh 2 mL microcentrifuge tubes (SARSTEDT AG & Co. KG, Nümbrecht, Germany) and stored at -20°C until batch analysis. All serum samples underwent a single freeze-thaw cycle, being thawed once for hormone and antibody level analysis.

Upon valid internal quality control analysis, the qualitative and quantitative determination of SARS-CoV-2 spike protein-specific IgG antibodies was conducted in accordance with the guidelines of “Laboratory Diagnostics for Coronavirus SARS-CoV-2” issued by the Austrian Society for Laboratory Medicine and Clinical Chemistry. External quality assessments provided by the Austrian Association for Quality Assurance and Standardization of Medical and Diagnostic Tests (ÖQUASTA) were implemented throughout the study duration.

Chemiluminescence immunoassays were performed to assess SARS-CoV-2 spike-protein-specific IgG using the VITROS Immunodiagnostic Products “Anti-SARS-CoV-2 IgG Quantitative assay” (including Quantitative Reagent Pack and Quantitative Calibrators) on the VITROS ECiQ Immunodiagnostic analytic instrument (Ortho Clinical Diagnostics GmbH, Germany). The measuring range of SARS-CoV-2-specific IgG antibodies ranged from 0 to 4000 BAU/mL.

### 2.3 Hormone analysis

DHEA, progesterone, testosterone, and cortisol were determined by a competitive enzyme-linked immunosorbent assay (ELISA) according to the manufacturer’s protocol (DRG Instruments GmbH, Marburg, Germany; DHEA ELISA reference no. EIA-3415, Progesterone ELISA reference no. EIA-1561, Testosterone ELISA reference no. EIA-1559, Cortisol ELISA reference no. EIA-1887). The inter-assay CV for the DHEA ELISA was determined in the scope of this study, since this ELISA used high and low controls. This value was calculated to be 10.0%. Samples were fully randomized prior to processing and singleton measurements were performed. The signal was read at 450 nm using a Tecan Infinite 200 plate reader (Tecan, Grödig, Austria).

### 2.4 Statistical analysis

The demographic information from the questionnaires and analytical findings were compiled into an Excel spreadsheet (version 1808, Microsoft, Redmond, WA, USA). Subsequently, the data underwent transfer and statistical analysis using IBM SPSS Statistics (version 29, Armonk, NY, USA) and GraphPad Prism (version 9.2.0, GraphPad Software, Boston, MA, USA) software.

The study population was stratified into two age groups: 19–40 years and ≥ 41 years. Participants aged over 60 were included in the ≥ 41 years group due to their low representation, thereby ensuring sufficient statistical power for analysis. Differences between age groups were analyzed using the Mann-Whitney test. To assess which factors affect the hormone level, multiple linear regression modeling was performed with hormone levels as a continuous dependent variable and by stepwise addition using p < 0.05 as the threshold for removing non-significant independent variables. Age, sex assigned at birth, body mass index (BMI), hormones, and disease were independent variables. Dummy variables were used for sex assigned at birth and disease (no chronic disease/chronic disease).

Levels of DHEA, cortisol, and testosterone were categorized into quartiles (Q) as following: DHEA Q1: 0.12 – 3.06 ng/mL, Q2: 3.07 – 4.63 ng/mL, Q3: 4.64 -7.16 ng/mL, Q4: 7.17 – 28.00 ng/mL; cortisol Q1: 0.00 – 84.42 ng/mL, Q2: 84.43 – 115.05 ng/ML, Q3: 115.06 – 156.52 ng/mL, Q4: 156.53 – 569.42 ng/mL; testosterone Q1: 0.04 – 0.56 ng/mL, Q2: 0.57 – 0.82 ng/mL, Q3: 0.83 – 3.80 ng/mL, Q4: 3.81 – 10.46 ng/mL. Progesterone was not categorized into quartiles since the distribution of the quartiles for progesterone did not allow for a reasonable interpretation of the data. Correlation analysis was performed using the Spearman Rho test. Groups below n = 3 were excluded from the statistical analysis.

### 2.5 Ethics

This research was conducted in accordance with the Declaration of Helsinki and approved by the Ethics Committee of Salzburg (EK-No. 1199/2021). Each subject agreed to participate in this study and signed an informed consent prior to any procedure. Participants’ anonymity was maintained throughout the whole study.

## 3 Results

**Figure 1:**
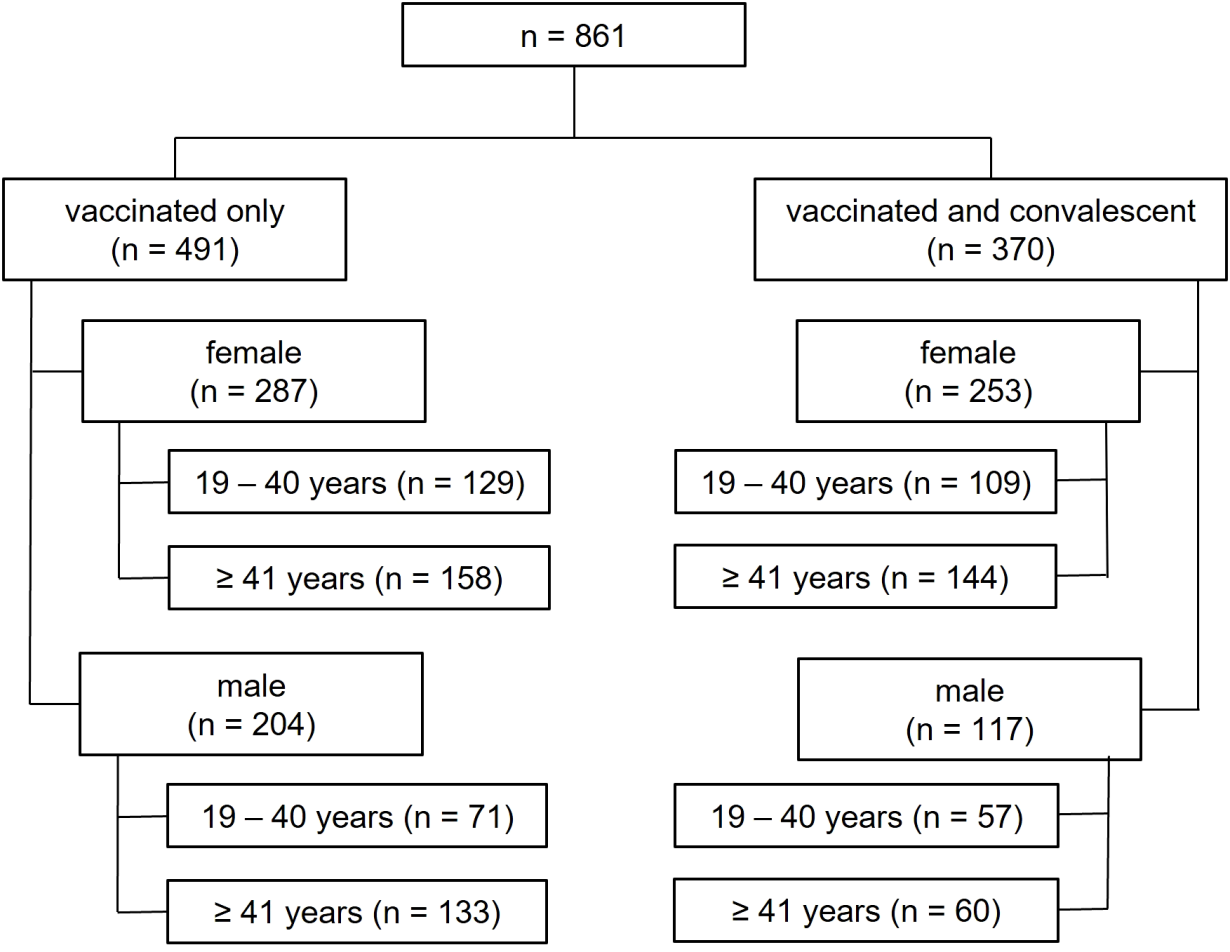
Analysis tree depicting the different study groups. The number of individuals in each group is shown in brackets.

The study population (n = 861) was divided into vaccinated only and hybrid immunized (vaccinated and convalescent). These two groups were further divided into sex assigned at birth, followed by 19–40 and ≥ 41 years of age. (**Figure 1**).

**Figure 2:**
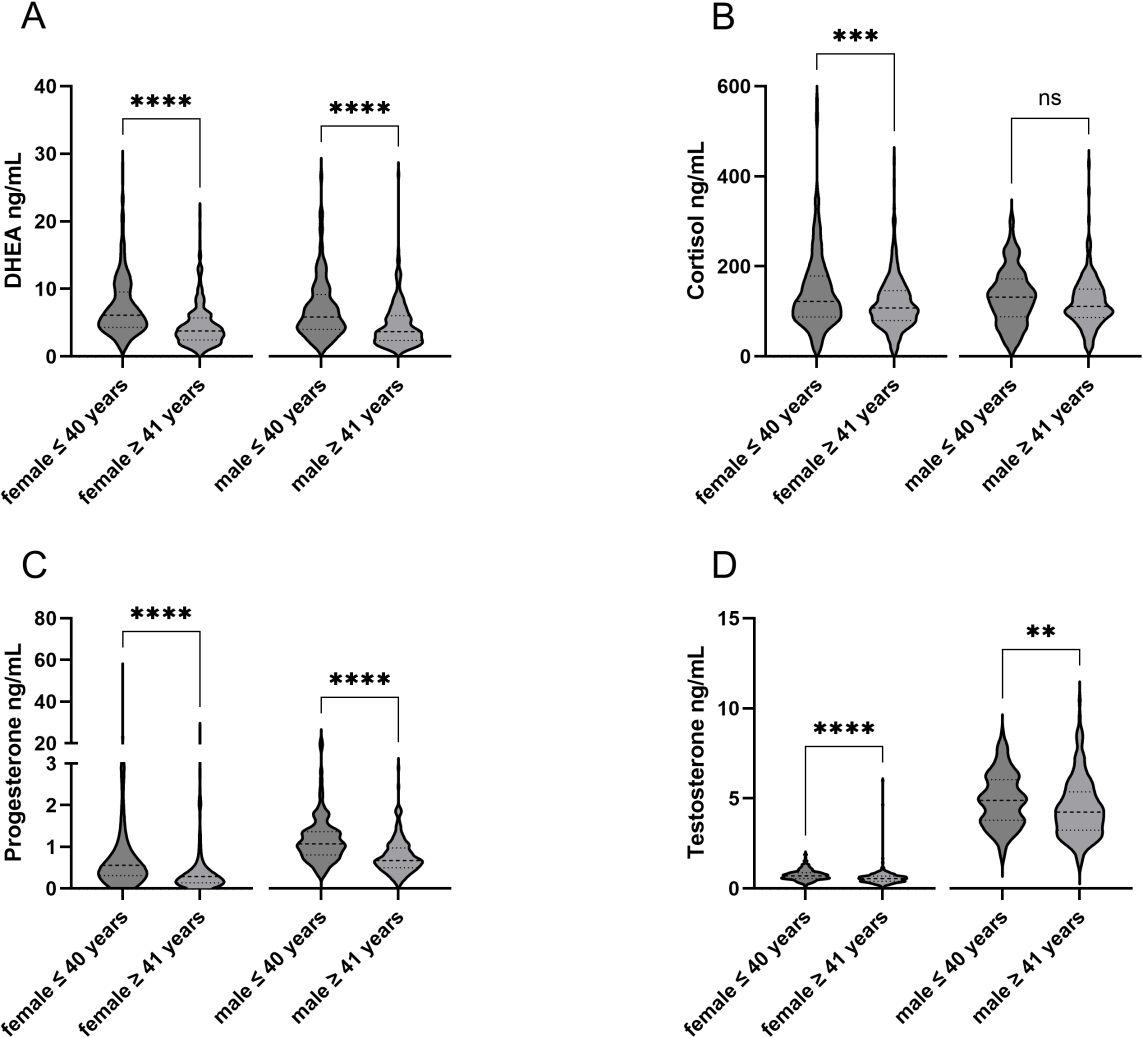
Comparison sex assigned at birth and age within hormones. Differences in the age groups can be seen within the respective hormones. **p-value < 0.01; ***p-value < 0.001; ****p-value < 0.0001. ns, not significant.

Of the 861 subjects, 62.7% were female and 37.3% were male with an average age of 42.9 ± 12.2 years (mean ± SD). The time between the last immunizing event and the blood draw was 11.0 ± 13.0 (median ± IQR) weeks within the overall study population. The demographics and characteristics of the study population are shown in **Table 1A**. Chronic diseases of the study population stratified into subpopulations are shown in **Supplementary Table S1**. The median sex hormone levels, stratified by sex assigned at birth and age, were calculated and are presented in **Table 1B**. The difference between the hormone levels regarding the age groups was analyzed within the female and male subject groups. Hormone levels of DHEA, cortisol, testosterone, and progesterone decreased significantly with age, except for cortisol in the male population (**Figure 2**). In addition, the distribution of hormones over age is shown in the **Supplementary Figure S1**.

**Table 1:** Demographics and median hormone levels of the study population.

A								
	Vaccinated only				Hybrid immunized			
	Female		Male		Female		Male	
Age (years)	≤ 40	≥ 41	≤ 40	≥ 41	≤ 40	≥ 41	≤ 40	≥ 41
Total n	128	158	73	131	110	144	55	62
BMI								
Underweight (< 18.5)	11	5	0	1	2	0	2	1
Normal (18.5 <=> 24.9)	84	87	45	43	75	88	31	26
Overweight (25.0 <=> 29.9)	20	52	19	62	26	34	19	23
Obese (> 30.0)	13	14	9	25	7	22	3	12
Chronic diseases								
Total n	24	53	7	46	23	55	9	24
Vaccines								
3 x Comirnaty	57	69	45	74	69	91	36	37
2 x Vaxzevria + 1 x Comirnaty	71	89	28	57	41	53	19	25
SARS-CoV-2 infection								
1 x	0	0	0	0	110	144	55	62
Time to last immunizing event								
Median ± IQR	17.4 ± 11.4	18.1 ± 8.5	17.9 ± 11.4	18.1 ± 12.0	7 ± 6.3	6 ± 6.0	6 ± 7.0	5 ± 4.7

B								
	Female (n = 540)				Male (n = 321)			
	19–40 years		≥ 41 years		19–40 years		≥ 41 years	
	(n = 238)		(n = 302)		(n = 128)		(n = 193)	
Sex hormone levels, median (IQR)								
Progesterone, ng/mL	0.56		0.29		0.5		0.31	
	(0.00 - 57.35)		(0.00 - 29.32)		(0.16 - 1.63)		(0.03 - 1.34)	
DHEA, ng/mL	6.1		3.79		5.85		3.69	
	(1.32 - 28.00)		(0.12 - 21.19)		(1.26 - 26.64)		(0.66 - 26.94)	
Cortisol, ng/mL	122.3		107.62		131.66		111.41	
	(17.05 - 569.42)		(0.00 - 434.87)		(13.34 - 304.95)		(16.09 - 427.35)	
Testosterone, ng/mL	0.71		0.55		4.89		4.24	
	(0.04 - 1.90)		(0.05 - 5.98)		(1.70 - 8.62)		(1.28 - 10.46)	

(A) Demographics and characteristics of the study population, stratified into subpopulations. Sample sizes (n) are reported unless specified differently. (B) The median hormone levels of 861 participants, stratified by sex assigned at birth and age.

Reference ranges of the hormones as stated by the ELISA manufacturer are shown in **Supplementary Table S2**. The median hormone levels of the subjects within this study align with the reference concentration ranges of the hormones. Some participants exhibited hormone levels exceeding the reference limit (**Table 1**). Therefore, statistical analysis was performed to investigate correlations between hormone levels and reported chronic diseases, but no significant correlation was observed (data not shown).

To examine which factors can potentially affect the hormone level in the cohorts vaccinated and hybrid immunized, a multiple linear regression analysis was performed using stepwise entry criteria with p < 0.05. The factors sex assigned at birth, age, titer, BMI, disease, and hormones were incorporated. The parameters that most frequently showed a significant influence on hormone levels were age and sex assigned at birth, followed by BMI and other hormone levels. Therefore, further analysis on the correlation of hormones and SARS-CoV-2-specific IgG titers were performed in groups stratified by sex assigned at birth and by age (19–40 years and ≥ 41 years) within the vaccinated and hybrid immunized cohorts. **Supplementary Tables S3** and **S4** show specific factors significantly affecting the hormone levels.

To investigate if hormones correlate with SARS-CoV-2-specific IgG titers, a Spearman Rho correlation analysis was performed, and all significant results are summarized in **Table 2**. For the hormone cortisol, a significant negative correlation was observed within the overall group of vaccinated male subjects (-0.210 *r*; p = 0.003) with an even stronger correlation within the subgroup of 19–40 years (-0.442 *r*; p < 0.001). Comparable findings were noted within the female hybrid overall group (-0.185 *r*; p = 0.003), with a more pronounced correlation value within the subgroup of 19–40 years (-0.229 *r*; p = 0.017). To further dissect this influence, the hormone concentration was split into quartiles and correlated to the antibody titer. Vaccinated females ≥ 41 years showed the highest negative correlation between cortisol in Q3 and antibody titers (-0.387 *r*; p = 0.031; Q3).

**Table 2:** Results of the Spearman Rho correlation (titer vs. hormone) within the respective groups.

Age groups	n	Hormone	Correlation (*r*)		Significance (*p*)
Vaccinated males					
Overall group	200	cortisol	-0.210	↓	0.003
	57	DHEA Q1	0.294	↑	0.026
19–40 years	52	testosterone Q4	-0.316	↓	0.023
	70	cortisol	-0.442	↓	< 0.001
	71	progesterone	-0.265	↓	0.026
≥ 41 years	52	DHEA Q1	0.273	↑	0.050
Vaccinated females					
Overall group	287	progesterone	-0.140	↓	0.017
≥ 41 years	31	cortisol Q3	-0.387	↓	0.031
	158	progesterone	-0.159	↓	0.047
Hybrid immunized females					
Overall group	107	testosterone Q2	0.218	↑	0.024
	253	cortisol	-0.185	↓	0.003
	253	progesterone	-0.148	↓	0.019
19–40 years	109	cortisol	-0.229	↓	0.017
≥ 41 years	144	progesterone	-0.180	↓	0.031

Only statistically significant groups are shown.Correlations are depicted using directional arrows and a color code for clarity: negative correlations are shown in blue, and positive correlations in red.

A significant positive correlation was seen for the hormone DHEA only in Q1 within the overall group of vaccinated males (0.294 *r*; p = 0.026) and the group of vaccinated males ≥ 41 years (0.273 *r*, p = 0.050), however no correlation was seen for the female subjects. In contrast, the hormone progesterone showed a significant negative correlation within the female subjects in both vaccinated (-0.140 *r*; p = 0.017) and hybrid groups (-0.148 *r*; p = 0.019) as well as in the ≥ 41 years group for vaccinated (-0.159 *r*; p = 0.047) female subjects. For the male population a significant negative correlation was only observed within the vaccinated male’s subgroup of 19–40 years (-0.265 *r*; p = 0.026).

Interestingly, the hormone testosterone showed a significant negative correlation within the vaccinated male’s subgroup of 19–40 years (-0.316 *r*; p = 0.023; Q4). However, a significant positive correlation within the female hybrid subjects (0.218 *r*; p = 0.024; Q2) was found.

To graphically demonstrate the correlation of SARS-CoV-2-specific IgG and hormone levels in different subject groups, scatter diagrams were generated (**Supplementary Figure S2**).

## 4 Discussion

Recent research has focused on identifying factors influencing SARS-CoV-2-specific antibody development, with implications for personalized COVID-19 prevention and management. Our prior study demonstrated that variables such as the type of vaccination, vaccine combinations, chronic illnesses, and certain medications significantly shape the humoral immune response to the virus (15).

A poorly understood factor is the potential modulatory role of hormones regulating immune function even though studies revealed significant gender differences in the severity and fatality of COVID-19 (7–9). To the best of our knowledge, this is the first comprehensive study to assess the association between SARS-CoV-2-specific antibody titers and the simultaneous measurement of four hormones (DHEA, cortisol, testosterone, and progesterone) in a large study cohort. This study aims to elucidate the role of hormones in the immune response to SARS-CoV-2.

First, we observed a positive correlation between DHEA levels and antibody titers in both the overall vaccinated male cohort and in males aged ≥ 41 years, using the lower quartile of DHEA levels as a reference. Notably, DHEA levels were not found to be a significant predictor of antibody titers within the female study cohort, highlighting a gender-specific difference in the immune response. These findings are not in line with a longitudinal observational cohort study conducted by Parthymou et al., which investigated the association between DHEA and SARS-CoV-2-specific antibody titers. In this study, 712 adults were tested for SARS-CoV-2-specific antibodies three months after vaccination with the Comirnaty vaccine (BioNTech and Pfizer). The authors of this study found no linear trend between antibody titers and DHEA, even when the latter was categorized into quartiles (16). Discrepancies may arise from differences in study design, as our data suggests that analyzing the cohort as a whole may mask important relationships. Subdividing the cohort into relevant subgroups, including division by sex and age, provided more insight into the relationship between DHEA and antibody titers.

Furthermore, our study showed that cortisol was negatively correlated with SARS-CoV-2-specific antibody titers in both sexes, suggesting that dysregulation of the hypothalamic-pituitary-adrenal (HPA) axis and excessive glucocorticoid signaling may be key mechanisms underlying impaired humoral immunity in the context of COVID-19. This aligns with prior research linking high cortisol to adverse COVID-19 outcomes and provides further evidence that modulating stress hormone pathways could be a promising therapeutic target (17, 18). Moreover, a negative correlation between BMI and cortisol as well as BMI and testosterone was found in both, vaccinated only and hybrid immunized group. This aligns with previous studies suggesting BMI as an influencing factor of testosterone and cortisol (19, 20).

We found divergent relationships between testosterone and antibody levels, with a negative correlation in vaccinated males aged 19–40 years (when the highest testosterone quartile was used as the reference) and a positive correlation in the hybrid-immunized female group (when the second quartile of testosterone levels was used as the reference). These sex-specific differences underscore the critical role of sex hormones in shaping the antibody response which may explain the well-documented disparities in COVID-19 susceptibility and severity between men and women (8, 21). This is supported by a prior study in male mice that found testosterone supplementation to improve serum IgG isotypes (22).

There was a significant negative correlation between progesterone and SARS-CoV-2 antibody titers in vaccinated males aged 19–40 years. In the female cohort, the interpretation of the progesterone findings was limited by the lack of data on the menstrual cycle phase at the time of blood collection. Nevertheless, a significant, however weak negative correlation between progesterone and SARS-CoV-2-specific IgG was observed in both vaccinated and hybrid-immunized females. The exact effects of progesterone on the immune system are currently not unambiguously described, with some studies suggesting it can promote T-helper cells type 2 cytokine production and IgE antibody formation, while others show that it reduces inflammatory responses and immune cell activation (14, 23, 24). This contradictory data forms the basis for further research.

An additional factor to consider is the time between the last immunizing event and the blood collection, which had a median of 11.0 weeks across our study cohort. Previous analyses in this cohort have shown that IgG antibody levels decline over time in both vaccinated and hybrid immunized subjects, but the decrease is more subtle in those with hybrid immunity (15). However, this temporal variable does not affect the observed hormone-antibody relationships within this study, since both values were derived from the same timepoint. Future studies assessing hormone status at the time of vaccination and correlating it with peak IgG antibody titers post-vaccination could provide additional valuable insights into the relationship between hormonal levels and antibody production.

Our analysis revealed that hormone levels varied widely, even within our otherwise healthy study population. This suggests that some individuals may have hormone concentrations that are not within the normal healthy range.

While our study provides important new insights into the relationship between hormone levels and SARS-CoV-2-specific IgG, there are some limitations that should be acknowledged. First, more female (62.7%) than male (37.3%) persons participated in this study, suggesting that women may be more willing to engage in such research. However, this gender disparity poses limitations on the generalizability of the findings. Second, the study did not account for circadian rhythms and other physiological conditions that can impact hormone levels, as blood draws were not performed at consistent times of the day. Single blood sampling represents a limitation to our study design and future studies would benefit from involving multiple samples per individual to capture both fluctuations and average levels. The use of singleton measurements (single determinations) rather than duplicate or triplicate assays represents a departure from standard research practice and reduces measurement precision. While this approach allowed analysis of a larger sample size within budget constraints, it introduces greater measurement uncertainty and should be considered when interpreting results.

Additionally, the measurement range for SARS-CoV-2-specific IgG antibodies was capped at 4000 BAU/mL, which may have limited the ability to detect associations at the higher end of the antibody titer spectrum. Furthermore, the interpretation of the progesterone findings in the female cohort was constrained by the lack of data on menstrual cycle phase at the time of blood collection. Fluctuations in progesterone levels over the course of the menstrual cycle are known to impact immune function (14), and this uncontrolled variable represents an important limitation of the present analysis. Although various common chronic diseases were assessed as potential influencing factors, no significant associations were observed. This may be attributed to the limited number of participants within each subgroup of chronic illnesses, which reduces statistical power. Further studies with larger subgroup sample sizes are recommended to better evaluate potential effects.

Future research would benefit from a more balanced gender distribution, stringent control of sampling time, extended antibody measurement ranges, and detailed tracking of menstrual cycle phase. Additionally, longitudinal monitoring of hormone levels and antibody titers, particularly post-vaccination, could provide more dynamic insights into their interplay. The incorporation of questionnaires assessing lifestyle factors and stress levels may also further improve control over potential confounding variables. Nonetheless, the large, diverse nature of our study population and the novel insights generated into sex-specific hormone-antibody relationships represent important strengths that help advance the field.

Our findings demonstrated that hormonal factors are associated with modulating the antibody response to SARS-CoV-2, highlighting a previously underappreciated aspect with important implications for personalized approaches to vaccination and treatment. By addressing this significant gap in literature, our research provides important new insights into the complex interplay between the endocrine and immune systems in COVID-19.

## Data Availability

The raw data supporting the conclusions of this article will be made available by the authors, without undue reservation.
