# Supplementary material for: Correlation between SARS-CoV-2-specific antibody titers and the hormones DHEA, cortisol, testosterone, and progesterone
Source: Front Immunol. 2025 Jul 31;16:1560623. doi: 10.3389/fimmu.2025.1560623 (PMC12350338; doi:10.3389/fimmu.2025.1560623)
Supplement: Supplementary file 1 [file DataSheet1.pdf]

# *Supplementary Material*

A

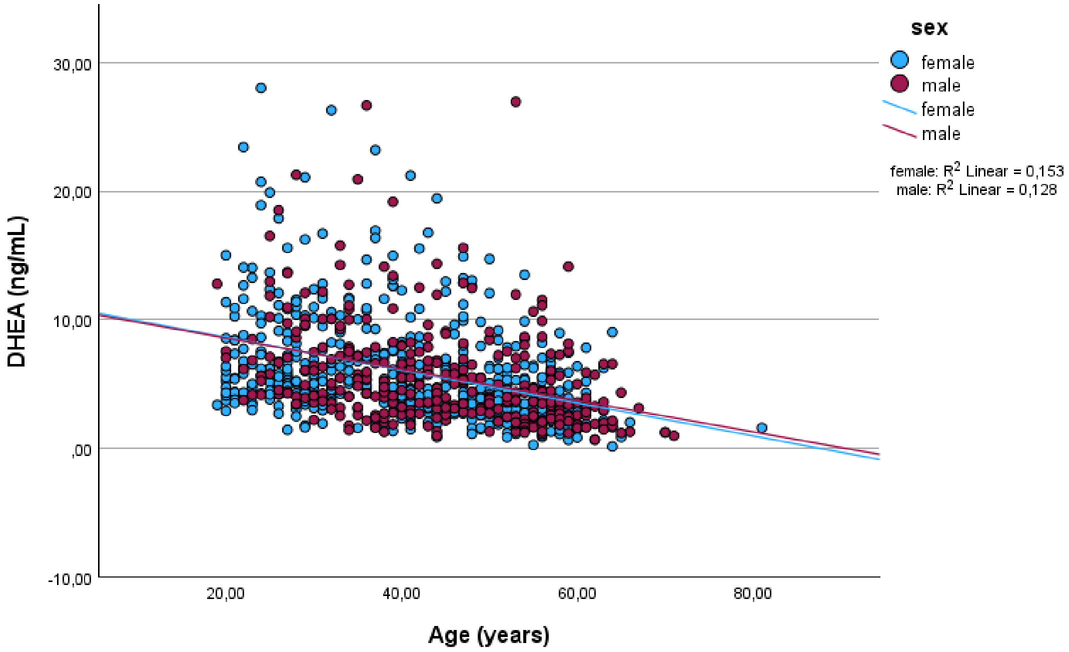


B

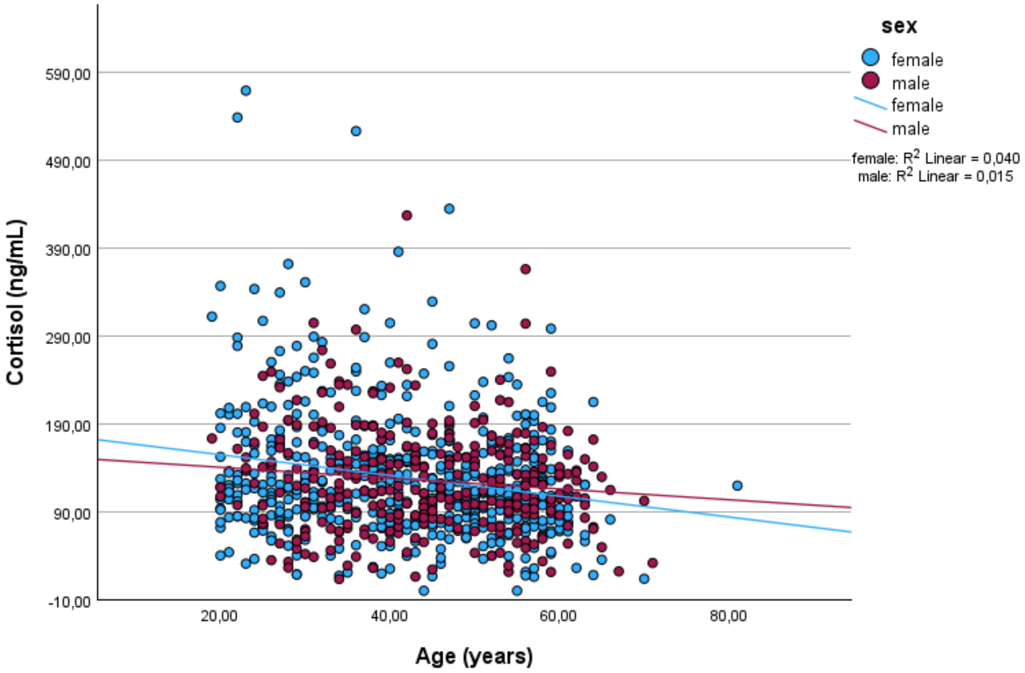

C

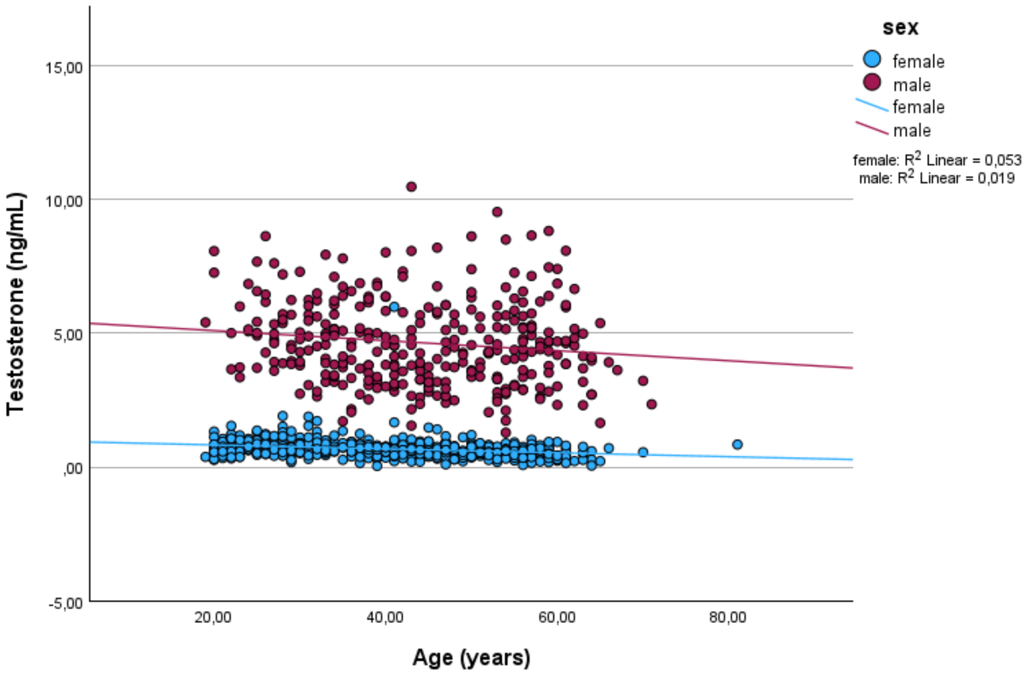


D

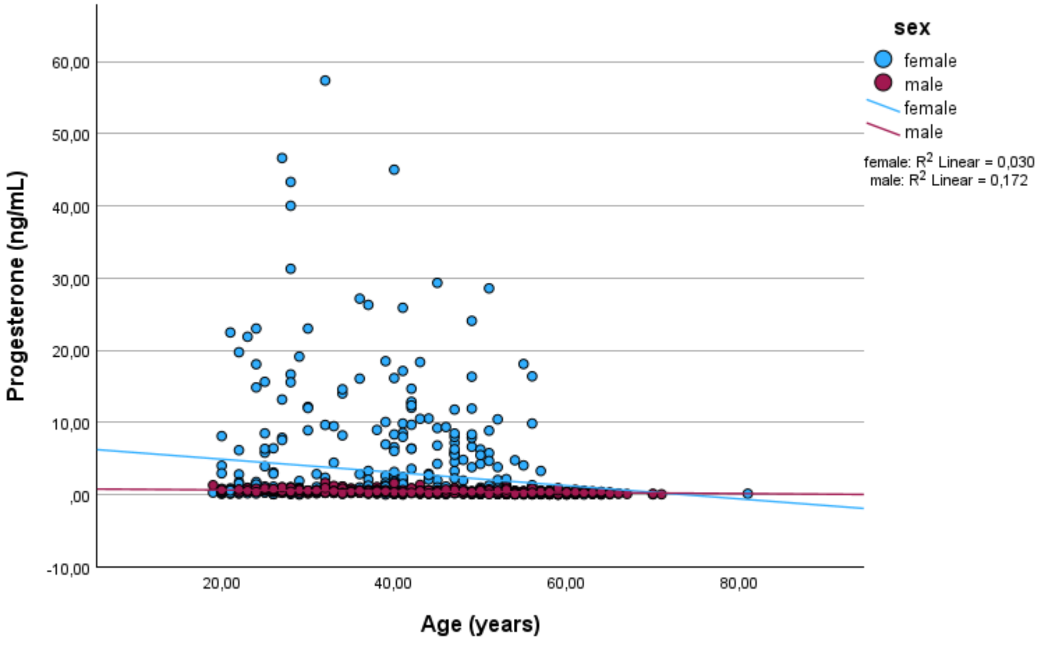


**Figure S1.** Scatter plots illustrate the distribution of hormones plotted against age within the whole study population (n = 861). (A) DHEA, Spearman's r = -0.452, p < 0.001 (female), r = -0.430, p < 0.001 (male); (B) cortisol, Spearman's r = -0.173, p < 0.001 (female), r = -0.117, p = 0.038 (male); (C) testosterone, Spearman's r = -0.365, p < 0.001 (female), r = -0.143, p = 0.010 (male); (D)

progesterone, Spearman's r = -0.400, p < 0.001 (female), r = -0.452, p < 0.001 (male). Blue dots indicate female sex (n = 540) and red dots male sex (n = 321). Linear regression line was added for male and female subject.

A

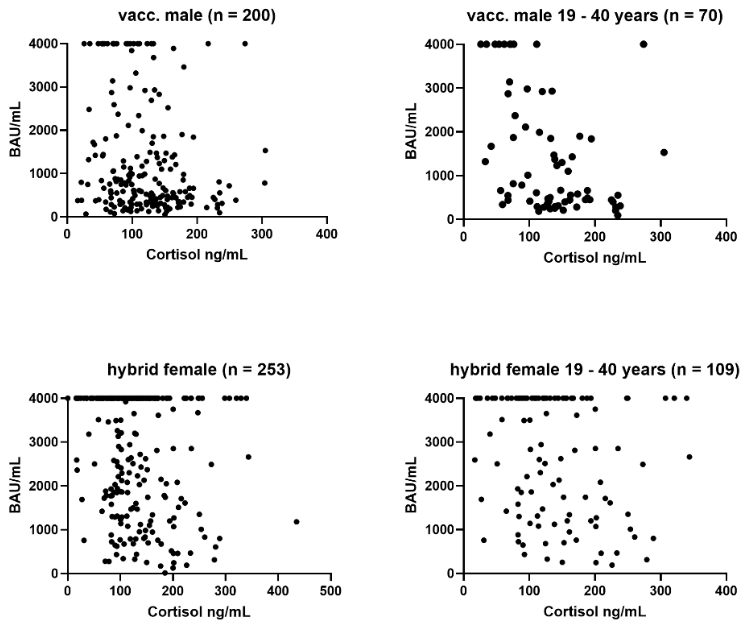


B

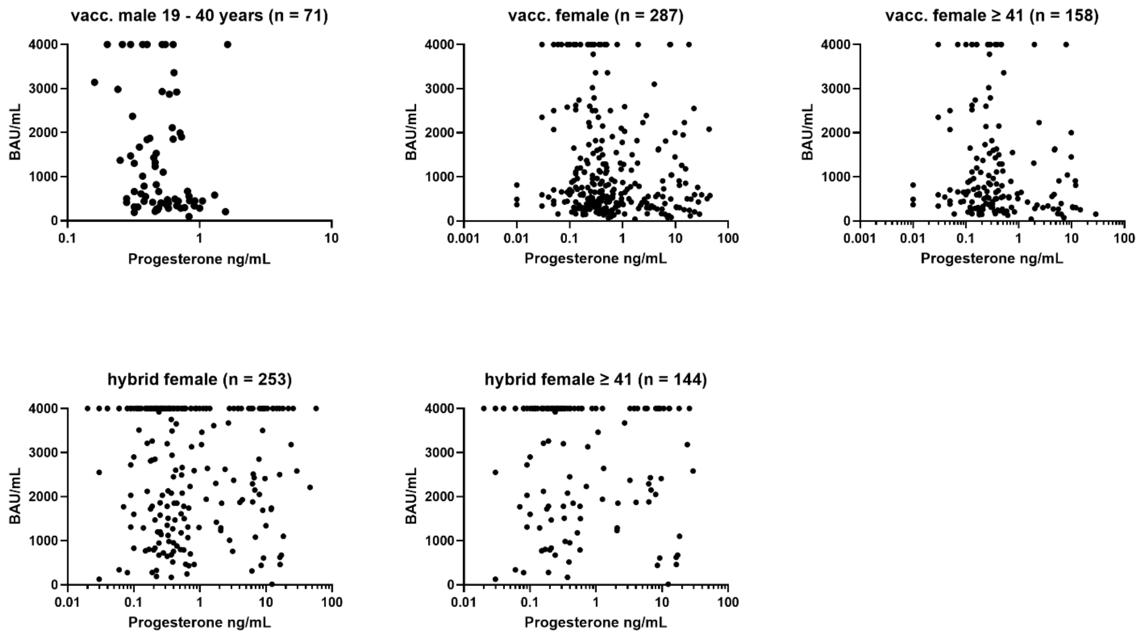

C

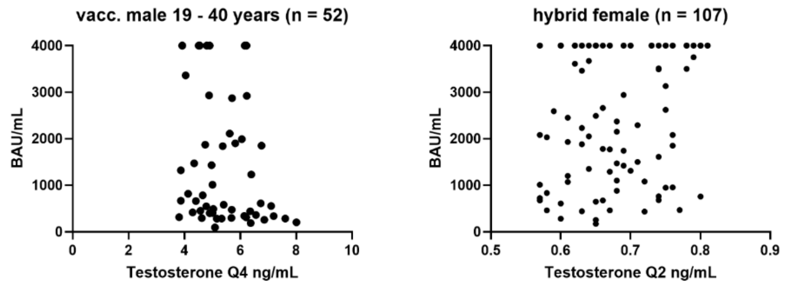


D

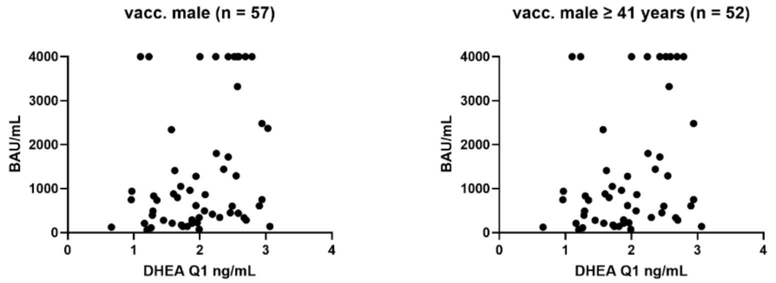


**Figure S2.** Correlation of the antibody level (BAU/mL) and the hormone levels (ng/mL) (A) cortisol, (B) progesterone, (C) testosterone, and (D) DHEA represented in a scatter diagram of statistically significant groups. Due to data distribution of the hormone progesterone, a log10 scale was used for better representation of the data. The limit of detection for SARS-CoV-2 antibodies is 4000 BAU/mL.

**Table S1.** Chronic diseases of the study population stratified into subpopulations. Sample sizes (n) are reported unless specified differently.

| | **Vaccinated only** | | | | **Hybrid immunized** | | | |
|---|---|---|---|---|---|---|---|---|
| | Female | | Male | | Female | | Male | |
| Age (years) | ≤ 40 | ≥ 41 | ≤ 40 | ≥ 41 | ≤ 40 | ≥ 41 | ≤ 40 | ≥ 41 |
| Total n | 128 | 158 | 73 | 131 | 110 | 144 | 55 | 62 |
| **Chronic diseases** | | | | | | | | |
| Colitis ulcerosa | 0 | 1 | 0 | 0 | 0 | 0 | 0 | 0 |
| High blood pressure | 1 | 10 | 2 | 22 | 1 | 8 | 0 | 13 |
| Psoriasis arthritis | 0 | 2 | 0 | 0 | 1 | 0 | 0 | 0 |
| Rheumatoide arthritis | 0 | 1 | 0 | 0 | 1 | 2 | 0 | 0 |
| Diabetes mellitus type 1 | 0 | 0 | 0 | 2 | 0 | 0 | 0 | 0 |
| Diabetes mellitus type 2 | 0 | 1 | 0 | 1 | 0 | 0 | 0 | 0 |
| Kidney disease | 0 | 0 | 0 | 1 | 0 | 0 | 0 | 0 |
| Liver disease | 0 | 0 | 1 | 0 | 0 | 0 | 0 | 0 |
| Thyroid disease | 12 | 20 | 0 | 5 | 7 | 23 | 2 | 3 |
| Other / multiple | 11 | 18 | 4 | 15 | 13 | 22 | 7 | 8 |

**Table S2.** Normal ranges according to the manuals of the ELISA used (DRG Instruments GmbH, Marburg, Germany).

| | **Female** | **Male** |
|---|---|---|
| Progesterone, ng/mL | 0.21 - 1.80 (follicular phase)<br>2.90 - 27.10 (luteal phase)<br>0.15 - 0.84 (postmenopausal) | 0.05 - 0.94 |
| DHEA, ng/mL | 0.12 - 11.55 | 0.83 - 18.09 |
| Cortisol, ng/mL | 50.00 - 230.00 (between 8:00 to 10:00 a.m.)<br>30.00 - 150.00 (at 4:00 p.m.) | |
| Testosterone, ng/mL | 0.26 - 1.22 | 2.00 - 6.90 |

**Table S3.** Final multivariable linear regression model for hormone levels – group vaccinated.

| Variable | Beta | P value |
| --- | --- | --- |
| **Dependent variable testosterone (adjusted $R^2 = 0.786$)** | | |
| Sex (female) | -0.899 | < 0.001 |
| DHEA (ng/mL) | 0.130 | < 0.001 |
| BMI | -0.086 | < 0.001 |
| **Dependent variable DHEA (adjusted $R^2 = 0.257$)** | | |
| Age | -0.321 | < 0.001 |
| Cortisol (ng/mL) | 0.241 | < 0.001 |
| Testosterone (ng/mL) | 0.413 | < 0.001 |
| Sex (female) | 0.345 | < 0.001 |
| **Dependent variable cortisol (adjusted $R^2 = 0.123$)** | | |
| DHEA (ng/mL) | 0.338 | < 0.001 |
| BMI | -0.092 | 0.032 |
| **Dependent variable progesterone (adjusted $R^2 = 0.086$)** | | |
| Sex (female) | 0.207 | < 0.001 |
| Age | -0.190 | < 0.001 |

**Table S4.** Final multivariable linear regression model for hormone levels – group hybrid immunized.

| **Variable** | **Beta** | **P value** |
|---|---|---|
| **Dependent variable testosterone (adjusted $R^2$ = 0.814)** | | |
| Sex (female) | -0.905 | < 0.001 |
| Age | -0.071 | 0.002 |
| BMI | -0.045 | 0.049 |
| **Dependent variable DHEA (adjusted $R^2$ = 0.266)** | | |
| Cortisol (ng/mL) | 0.372 | < 0.001 |
| Age | -0.318 | < 0.001 |
| **Dependent variable Cortisol (adjusted $R^2$ = 0.188)** | | |
| DHEA (ng/mL) | 0.406 | < 0.001 |
| BMI | -0.148 | 0.002 |
| **Dependent variable progesterone (adjusted $R^2$ = 0.038)** | | |
| Sex (female) | 0.201 | < 0.001 |
